# Meta Variational Memory Transformer for Anomaly Detection of Multivariate Time Series

**DOI:** 10.3390/s25247611

**Published:** 2025-12-15

**Authors:** Kun Qin, Yuxin Li, Wenchao Chen, Xinyue Hu, Bo Chen, Hongwei Liu

**Affiliations:** 1 Shanghai Aerospace Electronic Communication Equipment Institute, Shanghai 201109, China; kunq2025@163.com; 2 National Key Laboratory of Radar Signal Processing, Xidian University, Xi’an 710071, China; chenwenchao@xidian.edu.cn (W.C.); 24021110909@stu.xidian.edu.cn (X.H.); bchen@mail.xidian.edu.cn (B.C.); hwliu@xidian.edu.cn (H.L.)

**Keywords:** anomaly detection, memory networks, Bayes neural network

## Abstract

Detecting anomalies in multivariate time series (MTS) is a crucial task in areas like financial fraud detection and industrial equipment monitoring. Recent research has focused on developing unsupervised probabilistic models to identify anomalous patterns within MTS. However, many of these methods rely on fixed parameter mappings for each MTS, resulting in high computational costs and limited adaptability. To overcome these challenges, we introduce a novel **M**eta **V**ariational **M**emory **T**ransformer (MVMT). MVMT captures the diverse patterns across various MTS by encoding them into a set of memory units using a specially developed meta memory attention (MMA) module. Utilizing these learned memory units, we introduce a memory-guided probabilistic generative model that selects relevant memories as priors for latent states, resulting in more expressive MTS representations. A key feature of MVMT is that MMA provides a diversified prior in the latent space, ensuring the generation of various patterns. Finally, we implement a Transformer-based upward–downward variational inference process to estimate the posterior distribution of latent variables. Our extensive experiments on six datasets demonstrate the effectiveness of MVMT in one-for-all anomaly detection tasks.

## 1. Introduction

Anomaly detection in MTS stands as a critical scenario in Information Technology (IT) operations, particularly vital for financial fraud detection and industrial equipment monitoring, such as server machines within cloud infrastructures [1,2,3], content delivery networks [4], and more. Traditional statistical techniques are effective in simpler cases, such as univariate time-series anomaly detection [5,6,7,8,9]. However, their effectiveness may wane in more intricate and massive MTS scenarios. Consequently, attention has shifted towards machine learning-driven anomaly detection techniques, which are generally categorized into supervised learning approaches [5,10,11] and unsupervised methods [1,2,8,12,13,14,15,16].

Since anomalies are often infrequent and hidden within large volumes of normal data, identifying and labeling them is both challenging and costly. Moreover, supervised anomaly detection models often suffer from suboptimal performance due to severe class imbalance [17,18]. Consequently, unsupervised anomaly detection has gained significant traction in recent years. A major obstacle in unsupervised anomaly detection lies in effectively modeling the normal patterns in MTS. Among the various unsupervised methods, probabilistic dynamical models like OmniAnomaly [1] and SDFVAE [4] have excelled by capturing temporal correlations and variations inherent in time series. The emergence of Transformer architectures [19] and their ability to capture long-range dependencies [20,21,22,23] have further motivated their adoption for time-series modeling, and recent Transformer-based anomaly detectors [24] have shown strong representational capability.

**Figure 1:**
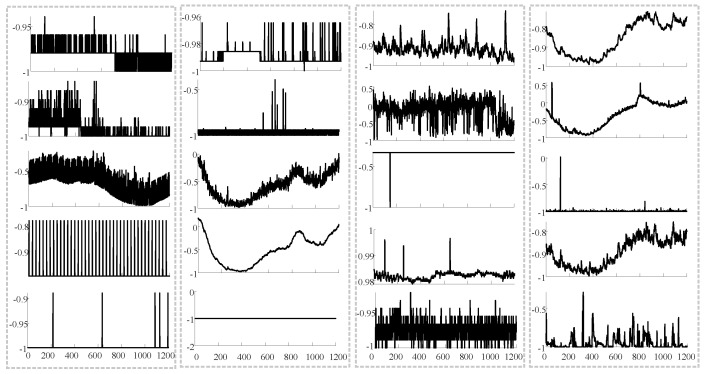
Visualization of pattern changes in five randomly selected channels from the SMD dataset at different time points. The figure highlights the pattern changes in five fixed channels across four different time points. Each column represents an MTS at a specific time, while each row corresponds to the same channel observed at different times. As shown in the figure, the same measured MTS can exhibit different patterns at different times, and these patterns may have significant distribution differences.

Since anomalies are often infrequent and hidden within large volumes of normal data, identifying and labeling them is both challenging and costly. Moreover, supervised anomaly detection models often suffer from suboptimal performance due to severe class imbalance [17,18]. Consequently, unsupervised anomaly detection has gained significant traction in recent years. A major obstacle in unsupervised anomaly detection lies in effectively modeling the normal patterns in MTS. Among the various unsupervised methods, probabilistic dynamical models like OmniAnomaly [1] and SDFVAE [4] have excelled by capturing temporal correlations and variations inherent in time series. The emergence of Transformer architectures [19] and their ability to capture long-range dependencies [20,21,22,23] have further motivated their adoption for time series modeling, and recent Transformer-based anomaly detectors [24] have shown strong representational capability. In contrast to physics-embedded MTS models [25], which rely on physical constraints or structure-preserving mappings, our method adopts a purely data-driven probabilistic framework. Rather than enforcing physics-guided latent spaces, we introduce a meta memory prior to capture diverse temporal patterns across heterogeneous MTSs, making the approach suitable when explicit physical laws are unavailable. However, these models typically learn a single shared representation and lack an explicit mechanism to encode and differentiate the diverse normal patterns that arise across heterogeneous MTS sources. As illustrated in Figure 1, different devices or subsystems may exhibit fundamentally different operational modes, making it difficult for a single Transformer encoder to generalize. Without structural priors, attention alone must implicitly capture all such variations, which often leads to underfitting of rare-but-normal behaviors and reduced robustness when intra-class variability is high [26]. These limitations motivate the introduction of a meta memory prior that can store, organize, and retrieve prototypical temporal patterns learned across multiple MTSs. By providing diversified and informative priors for latent states, such a mechanism fills the gap left by existing Transformer-based anomaly detectors and enables more expressive and adaptable representations in a unified one-for-all anomaly detection framework.

**Figure 2:**
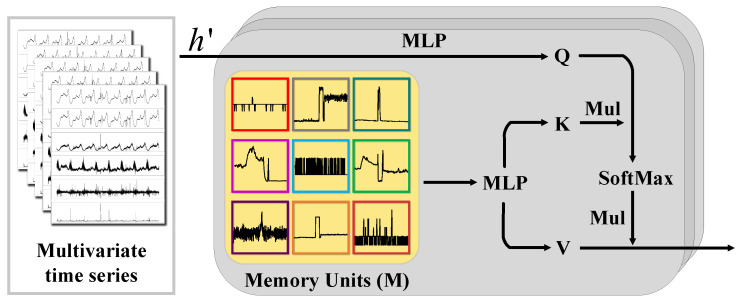
Meta Memory Attention (MMA). We constructed this module by incorporating a multi-headed attention mechanism. This approach significantly enhances our model’s memory recall capability.

To overcome the aforementioned limitations, we introduce an innovative memory-guided probabilistic generative model tailored to capture intricate and diverse temporal dependencies across multiple MTSs. Our approach draws inspiration from meta-learning [27], aiming to extract transferable patterns across related tasks. We devised the Meta Memory Attention (MMA) mechanism to identify and retain complex and diverse temporal information shared among multiple MTSs, storing it within the memory units as illustrated in Figure 2. Subsequently, we developed a memory-guided probabilistic generative model within a hierarchical Bayesian framework. This model leverages the diverse temporal dependencies by employing the stored memory from MMA as a prior, thereby facilitating the acquisition of expressive representations crucial for anomaly detection within MTS data. To optimize the inference process for our model, we implement an innovative upward-downward autoencoding variational inference strategy. This approach integrates both bottom-up likelihood assessments and top-down prior knowledge of parameters to achieve a more accurate posterior approximation. Additionally, we enhance the inference network by integrating an anomaly transformer, which refines the latent representations. The key contributions of our work are summarized as follows:

- To consider diverse nomal patterns within MTSs and achieve anomaly detection, we propose a hierarchical Bayesian network named MVMT. MVMT consists of a developed MMA module that captures and stores various temporal dependencies of MTSs, a memory-guided generative module to capture the shared diverse temporal information, as well as a transformer-powered inference module for accurate posterior approximation.
- We carefully design an upward–downward variational inference model built upon the transformer blocks for MVMT to approximate the posterior for the latent variables.
- While most existing methods rely on distinct parameter sets for each MTS, the proposed MVMT utilizes a single group of parameters to model diverse MTSs, allowing it to effectively capture multiple patterns within a unified framework.
- We perform thorough experiments on six real-world datasets. The quantitative comparison results demonstrate that MVMT surpasses state-of-the-art methods in terms of F1-score with fewer parameters. The qualitative analysis reveals that it effectively captures diverse dynamic memory patterns within different MTSs.

## 2. Related Work

Recently, unsupervised anomaly detection has become a focal point for operational engineers. Many of these methods adopt an encoding-reconstruction approach, leveraging architectures like AE, VAE, or GAN. To model the characteristics of MTS, these methods are typically based on either RNNs [28] or Transformers [19]. For instance, EncDec-AD [15] uses an LSTM-based AE architecture to capture temporal dependencies in MTS, detecting anomalies through deviations from reconstruction. Similarly, Telemanom [16] leverages LSTM to predict spacecraft telemetry channel values and detects anomalies by evaluating the residual error between predicted and observed values. Additionally, MAD-GAN [13] employs LSTM-RNN within a GAN to model typical temporal and spatial patterns. OmniAnomaly [1] uses a stochastic recurrent neural network (SRNN) to robustly learn representations, while SDFVAE [4] introduces a static and dynamic factorized VAE approach specifically addressing both time-invariant and time-varying elements. VAE-based models, such as OmniAnomaly and SDFVAE, detect anomalies by evaluating likelihood, with lower likelihood values suggesting a higher probability of an anomaly. It is important to highlight that these unsupervised approaches are largely RNN-based, concentrating on capturing temporal dependencies in MTSs. However, there is a recent inclination towards leveraging transformers, known for their efficiency in capturing intricate and long-range temporal dependencies, as well as in MTS anomaly detection [29]. Anomaly transformer [24] has emerged as the top performer in MTS anomaly detection thus far.

Although current unsupervised anomaly detection methods have shown effectiveness in practical applications, capturing diverse temporal dependencies across multiple MTSs remains a significant challenge. The common approach of training separate models for each MTS limits comprehensive modeling, leading to high computational costs and reduced adaptability. To address this issue, GmVRNN [30] seeks to handle diverse MTSs with a single set of parameters by using a Gaussian mixture distribution for latent states. Despite its promising results, this approach often struggles to capture all patterns effectively, as a single parameter set may not be sufficient, requiring adjustments for different MTSs to ensure optimal performance. This paper introduces MVMT, a pioneering Transformer-based probabilistic model. MVMT captures the diverse patterns across various MTS by encoding them into a set of memory units using a specially developed meta memory attention (MMA) module. Utilizing these learned memory units, we introduce a memory-guided probabilistic generative model that selects relevant memories as priors for latent states, resulting in more expressive MTS representations.

## 3. Preliminaries

This section presents a brief overview of the problem definition, the MVMT-based anomaly detection approach, and the data processing methodology. Additionally, we clarify the meanings of the symbols used in the paper, as detailed in Table 1.

### 3.1. Problem Definition

Consider the MTS as $X=\{x_{1},x_{2},...,x_{T_{l}}\}$, where $T_{l}$ represents the total length of X, and $x_{t}\in R^{V}$ is the observation at time *t*, with *V* indicating the dimensionality at each timestep. Thus, $X\in R^{T_{l}\times V}$. Anomaly detection in MTS involves determining whether a specific observation at a given time, denoted as $x_{t}$, is anomalous. In an unsupervised approach, the model is trained exclusively on normal data to capture normal patterns in MTS. During the testing phase, reconstruction errors are used as anomaly scores. The anomaly score function should produce higher values for anomalous MTS compared to normal ones.

### 3.2. Anomaly Detection Based on MVMT

**Figure 3:**
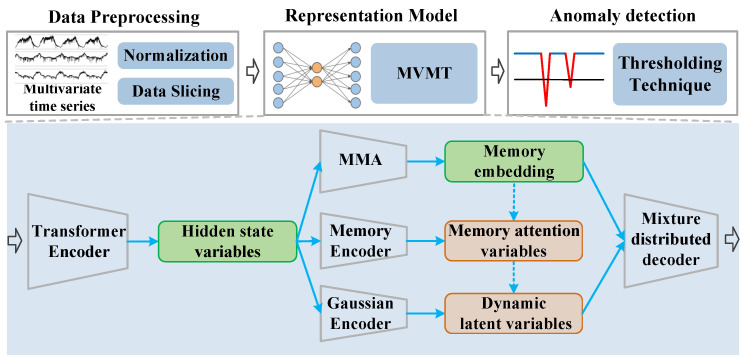
Overall framework of anomaly detection for MTS based on MVMT. Solid lines represent the inference process, whereas dashed lines denote the generation process.

The overarching structure for unsupervised anomaly detection utilizing MVMT is depicted in Figure 3. Our framework consists of three components: MTS preprocessing, representation model, and anomaly detection modules. Specifically, MTS preprocessing is introduced in Section 3.3, and MVMT is employed as the representation model. The reconstruction probability inferred by MVMT is utilized to guide the detection of anomalies.

As the model learns the typical patterns of normal MTS, observations that closely match these learned behaviors are reconstructed with high accuracy and confidence. Therefore, we use the reconstruction error as an anomaly score to assess deviations from expected behavior [1,2,8,31]. The anomaly score is computed as follows:

(1) $$\begin{array}{c} S_{t}=\log p\left(x_{t}|z_{t},m_{a}\right) \end{array}$$

where $S_{t}$ is the anomaly score, and $m_{a}$ and z are two components in the latent space of the proposed model, which will be explained in detail in Section 4. Because our model is built within a Bayesian probabilistic framework, the reconstruction term is naturally expressed as the log-likelihood. This choice ensures consistency with the hierarchical VAE derivation, whereas MSE residuals correspond to deterministic autoencoders and are not directly aligned with our probabilistic formulation. To classify an observation $x_{t}$ as anomalous, we compare its anomaly score $S_{t}$ against a predefined threshold. We use the POT method [32] to set this threshold. In MVMT, smaller anomaly scores are deemed more likely to indicate outliers. Consequently, similar to [1], we select thresholds based on lower-bound criteria to identify extreme values.

### 3.3. Data Preprocess

Similar to the approach in [33], for observed MTSs, we initially normalize them as

(2) $$x_{t}\leftarrow \frac{x_{t}-\min\left(X\right)}{\max(X)-\min(X)+\epsilon }$$

where max(X) and min(X) denote the maximum and minimum values for each feature across the training time series, respectively, and ε is a small constant (set to $10^{-6}$) introduced to avoid division-by-zero instability, and the results are insensitive to its value within a reasonable numerical range. After normalizing the MTSs, we employ a sliding window technique with overlap, following [4], for dataset preprocessing. Here, the window size T=20, and there is an overlap of l=T−1 between two consecutive observed variables. Notably, to capture both time-varying and time-invariant information, we augment each timestep with its *w* neighbors, set to 5 for anomaly detection tasks.

## 4. Methodology

In this section, we outline the key concepts of the proposed MVMT framework, followed by an in-depth discussion of the MMA model, including its generative and inference components. To facilitate understanding, Table 1 provides a summary of the symbols used throughout the paper.

### 4.1. Meta Variational Memory Transformer

**Figure 4:**
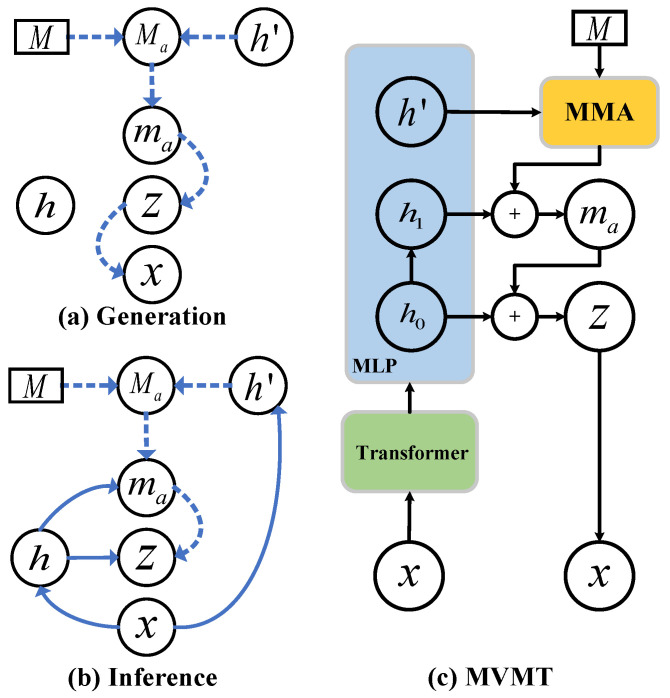
Graphical illustration of MVMT: (**a**) generation process of x; (**b**) inference of the variational distribution of $m_{a}$ and z; (**c**) overview of the proposed MVMT. Solid lines represent the inference process, whereas dashed lines denote the generation process.

As depicted in Figure 4, MVMT establishes a memory model based on the transformer architecture from a variational Bayesian perspective. Our approach introduces a latent space comprising two components: $m_{a}$ and z. To capture and retain diverse patterns, we devised the MMA module, constructed upon a set of memory units organized using the attention mechanism. $m_{a}$ serves as a memory prior for z, ensuring an expressive latent representation and facilitating the generation of MTS with diverse temporal information. In contrast to the conventional VAE [34], MVMT defines the latent variable z with a memory prior ($m_{a}$), integrating diverse temporal information from multiple MTSs. This structuring of the latent space is represented as follows in equation form:

(3) $$\begin{array}{c} p\left(x\right)=\int_{m_{a}}\int_{z}p\left(x|z,m_{a}\right)p\left(z|m_{a}\right)p\left(m_{a}\right)dm_{a}dz \end{array}$$

Based on the latent space defined in Equation (3), we present a refined MMA model alongside a Bayesian generative framework to effectively capture and represent the full latent space.

#### 4.1.1. Meta Memory Attention Module

Global diverse temporal information within multiple MTSs is crucial for modeling multiple MTSs with a single model [30]. Inspired by Concept Transformer (CT) [35], we aim to capture and store diverse temporal information within MTS through a meta memory module, employing an attention module to facilitate memory operations. Specifically, considering *N* memory units $M\in R^{N\times d_{m}}$ and MTS features $h^{\prime }\in R^{d_{m}}$, where $d_{m}$ represents the vector dimension, M consists of vectors optimized by a well-designed loss function proposed later. Subsequently, an attention mechanism is utilized to derive the memory embedding $M_{a}$. As illustrated in Figure 2, the input feature $h^{\prime }$ initially undergoes mapping via a MLP to generate the query matrix $Q\in R^{N\times d_{m}}$ for the query-key-value cross-attention module. Following this, the memory units M serve as input to predict the key matrix $K\in R^{N\times d_{m}}$ and value matrix $V\in R^{N\times d_{m}}$. The attention weights matrix $A=\left[\alpha \right]\in R^{N\times N}$ can be obtained from Q and K as

(4) $$\begin{array}{c} \alpha =\mathrm{softmax}\left(\frac{1}{\sqrt{d_{m}}}QK^{⊤}\right) \end{array}$$

Final result from the MMA is computed by applying the attention map A to the value matrix V, yielding the weighted output.

(5) $$\begin{array}{c} M_{a}=\sum_{i=1}^{N}{\left[AV\right]}_{i} \end{array}$$

As shown in Figure 4a, with the memory embedding ($M_{a}$) defined in Equation (5), we introduce a memory latent variable $m_{a}$:

(6) $$\begin{array}{cc} \; & p\left(m_{a}|M_{a}\right)∼N\left(m_{a}|\mu_{m_{a}},\mathrm{diag}\left(\sigma_{m_{a}}^{2}\right)\right)\\ \; & \mu_{m_{a}}=G_{\mu }^{M_{a}}\left(M_{a}\right)=f\left(W_{\mu }^{M_{a}}M_{a}+b_{\mu }^{M_{a}}\right)\\ \; & \sigma_{m_{a}}^{2}=G_{\sigma }^{M_{a}}\left(M_{a}\right)=f\left(W_{\sigma }^{M_{a}}M_{a}+b_{\sigma }^{M_{a}}\right) \end{array}$$

where $m_{a}$ is treated as a Gaussian random variable, $G_{\mu }^{M_{a}}$ and $G_{\sigma }^{M_{a}}$ are two functions used to generate $m_{a}$ from $M_{a}$. Specifically, we utilize a fully connected network as $G^{M_{a}}$, and f(·) represents a deterministic non-linear transition function, where $W^{M_{a}}\in R^{d_{m}\times d_{m}}$ denotes the transition matrices, and $b^{M_{a}}\in R^{d_{m}}$ represents the bias weights. From Equation (6), $m_{a}$ is conditioned on the memory embedding $M_{a}$. The advantage of the attention mechanism in MMA allows $m_{a}$ to provide powerful memory information for subsequent models. The memory units are initialized with small random values and are jointly updated with all model parameters through standard back-propagation under the variational objective, which ensures stable training and practical convergence.

#### 4.1.2. Probablistic Memory Generative Model

Figure 4a illustrates how we model the diverse temporal dependencies in MTS by utilizing the multiple patterns stored in the MMA module. To achieve this, we design a probabilistic memory generative model based on a hierarchical Bayesian framework [36], where the latent variable is defined as

(7) $$\begin{array}{cc} \; & p\left(z|m_{a}\right)∼N\left(z|\mu_{z},\mathrm{diag}\left(\sigma_{z}^{2}\right)\right)\\ \; & \mu_{z}=F_{\mu }^{m_{a}}\left(m_{a}\right)=f\left(W_{\mu }^{m_{a}}m_{a}+b_{\mu }^{m_{a}}\right)\\ \; & \sigma_{z}^{2}=F_{\sigma }^{m_{a}}\left(m_{a}\right)=f\left(W_{\sigma }^{m_{a}}m_{a}+b_{\sigma }^{m_{a}}\right) \end{array}$$

where $F_{\mu }^{m_{a}}$ and $F_{\sigma }^{m_{a}}$ are two functions used to generate z from $m_{a}$. Specifically, we employ a fully connected network as $F^{m_{a}}$, and f(·) represents a deterministic non-linear transition function. Here, $W^{m_{a}}\in R^{d_{m}\times d_{m}}$ denotes the transition matrices, and $b^{m_{a}}\in R^{d_{m}}$ represents the bias weights. Unlike traditional VAEs, where the latent variable z is typically conditioned on a standard Gaussian distribution, in our model, z is conditioned on the latent variable $m_{a}$. This enables MVMT to consider diverse temporal dependencies based on the meta-information stored in MMA. As depicted in Equation (6), $p(m_{a}|M_{a})$ provides globally diverse temporal information for each latent variable. Similar to [30], for considering the stochasticity within MTS, we assign the generated x to follow a Gaussian distribution, defined as

(8) $$\begin{array}{c} p\left(x|z\right)∼N\left(x|\mu_{x},diag\left(\sigma_{x}^{2}\right)\right) \end{array}$$

where $\mu_{x}$ and $\sigma_{x}^{2}$ are mean and variance parameters that are conditioned on the hidden variable z as

(9) $$\begin{array}{c} \mu_{x}=F_{\mu }^{z}\left(z\right)=f\left(W_{\mu }^{z}z+b_{\mu }^{z}\right)\\ \sigma_{x}=F_{\sigma }^{z}\left(z\right)=f\left(W_{\sigma }^{z}z+b_{\sigma }^{z}\right) \end{array}$$

$F_{\mu }^{z}$ and $F^{z}\sigma$ are two functions used to generate x from z. Similar to Equation (7), we utilize a fully connected network as $F^{m_{a}}$, where model parameters $W^{z}$ and $b^{z}$ have the same settings. This approach enables the model to transfer the diverse temporal information stored in the MMA from $m_{a}$ to z, ultimately aiding in reconstructing x. The hierarchical information generation allows the model to consider the diversity within multiple MTS and acquire more expressive and robust representations for them. This is essential for unsupervised anomaly detection methods to enhance their performance.

#### 4.1.3. Inference Model

As with most Bayesian models, directly computing the exact posterior distribution is intractable, a challenge common in VAEs [34,37]. To address this, we define the variational distribution $q(m_{a},z|x,M_{a})$, which must be sufficiently flexible to closely approximate the true posterior. To efficiently approximate the posterior for the hierarchically structured latent variables in MVMT, we design an inference network that integrates both bottom-up likelihood information and top-down prior information, allowing for a more accurate approximation of the generative process. The variational distribution is defined as

(10) $$\begin{array}{c} q\left(m_{a},z|M_{a},x\right)=q\left(m_{a}|M_{a},x\right)q\left(z|m_{a},x\right) \end{array}$$

Specifically, to capture the complex temporal characteristics of MTSs, we initially employ an anomaly-transformer-like block (using the model structure but without the minimax strategy proposed by [24]) to extract the features of MTSs, and the parameters of the block are defined as $W_{xh_{0}}$. As depicted in Figure 4c, for the upward flow of information, we compute the variable $h_{0}$ through the anomaly transformer and then utilize a fully connected network to obtain the variables $h_{1}$ and $h^{\prime }$ as follows:

(11) $$\begin{array}{c} h_{0}=\mathrm{Anomaly}\;\mathrm{Transformer}(x)\\ h_{1}=f\left(W_{h_{0}h_{1}}h_{0}+b_{h_{0}h_{1}}\right)\\ h^{\prime }=f\left(W_{h_{0}h^{\prime }}h_{0}+b_{h_{0}h^{\prime }}\right) \end{array}$$

As depicted in Figure 4b,c, we estimate $m_{a}$ by integrating the extracted latent features with prior knowledge from the MMA module, constructing the variational posterior for $m_{a}$ as follows:

(12) $$\begin{array}{cc} q(m_{a}|M_{a},h_{1}) & ∼N\left(m_{a}|{\tilde{\mu }}_{m_{a}},\mathrm{diag}\left({\tilde{\sigma }}_{m_{a}}^{2}\right)\right)\\ {\tilde{\mu }}_{m_{a}}=\Phi \left(M_{a},h_{1}\right) & =f({\tilde{W}}_{m_{a}}^{\mu }M_{a}+{\tilde{V}}_{m_{a}}^{\mu }h_{1}+{\tilde{b}}_{m_{a}}^{\mu })\\ {\tilde{\sigma }}_{m_{a}}=\Phi \left(M_{a},h_{1}\right) & =f({\tilde{W}}_{m_{a}}^{\sigma }M_{a}+{\tilde{V}}_{m_{a}}^{\sigma }h_{1}+{\tilde{b}}_{m_{a}}^{\sigma }) \end{array}$$

where $\{{\tilde{W}}_{m_{a}}^{\mu }$,${\tilde{V}}_{m_{a}}^{\mu }$, ${\tilde{W}}_{m_{a}}^{\sigma }$, ${\tilde{V}}_{m_{a}}^{\sigma }$ $\}\in R^{d_{m}\times d_{m}}$ and $\{{\tilde{b}}_{m_{a}}^{\mu }$, ${\tilde{b}}_{m_{a}}^{\sigma }\}$ $\in R^{d_{m}}$ are the parameters that the inference model learns during training. As a hierarchical VAE, the inference model requires separate latent flows: $h_{0}$ estimates the posterior of the lower-level latent variable z, while $h_{1}$ provides a higher-level approximation for $m_{a}$, ensuring that deeper latent layers are inferred from correspondingly more abstract features. This approach allows $m_{a}$ to be estimated by integrating both the bottom-up likelihood information present in $h_{1}$ from the inference network and the contextual memory from the MMA module. By combining these sources, the proposed inference network effectively captures long-range and diverse temporal dependencies, leading to a rich latent representation for MVMT. The latent variable $q(m_{a}|M_{a},h_{1})$ is then reparameterized as

(13) $$\begin{array}{c} m_{a}={\tilde{\mu }}_{m_{a}}^{k}+{\tilde{\sigma }}_{m_{a}}^{k}\epsilon ,\epsilon ∼\mathrm{Uniform}\left(0,1\right) \end{array}$$

which allows for back-propagation through z. MVMT combines the obtained latent features by the inference network with the prior from $m_{a}$ to construct the variational posterior as

(14) $$\begin{array}{cc} q(z|m_{a},h_{0}) & ∼N\left(z|{\tilde{\mu }}_{z},\mathrm{diag}\left({\tilde{\sigma }}_{z}^{2}\right)\right)\\ {\tilde{\mu }}_{z}=\Psi \left(m_{a},h_{0}\right) & =f({\tilde{W}}_{z}^{\mu }m_{a}+{\tilde{V}}_{z}^{\mu }h_{0}+{\tilde{b}}_{z}^{\mu })\\ {\tilde{\sigma }}_{z}=\Psi \left(m_{a},h_{0}\right) & =f({\tilde{W}}_{z}^{\sigma }m_{a}+{\tilde{V}}_{z}^{\sigma }h_{0}+{\tilde{b}}_{z}^{\sigma }) \end{array}$$

where $\{{\tilde{W}}_{z}^{\mu }$,${\tilde{V}}_{z}^{\mu }$, ${\tilde{W}}_{z}^{\sigma }$, ${\tilde{V}}_{z}^{\sigma }$ $\}\in R^{d_{m}\times d_{m}}$ and $\{{\tilde{b}}_{z}^{\mu }$, ${\tilde{b}}_{z}^{\sigma }\}$ $\in R^{d_{m}}$ denote the learnable parameters that map $m_{a}$ and $h_{0}$ to z. This allows z to be inferred by integrating the likelihood information from $h_{0}$ with the prior knowledge provided by $m_{a}$, thus facilitating the generation of rich latent representations for MVMT. Additionally, $q(z|m_{a},h_{0})$ can be reparameterized as

(15) $$\begin{array}{c} z={\tilde{\mu }}_{z}+{\tilde{\sigma }}_{z}\epsilon ,\epsilon ∼\mathrm{Uniform}\left(0,1\right) \end{array}$$

which facilitates the use of the back-propagation algorithm. We note that MVMT incorporates transformer structure into inference processes, which enables it to model complex temporal dependencies within MTS efficiently in both processes, thus guaranteeing expressive latent representation learning and promising anomaly detection performance.

### 4.2. Model Properties

MVMT leverages the advantages of the hierarchical Bayesian model and the meta memory model enhanced by the attention mechanism, as detailed below.

#### 4.2.1. Learnable Memory Prior

The general VAE [34] uses the standard normal distribution as the prior, which does not contain information. We designed the MMA to organize and store the information from the big data and select the corresponding memory for different MTS to generate a dedicated prior. This prior gives our model the ability to adapt quickly to the new MTS. Moreover, we designed an attention-based meta memory module to efficiently manage global information and provide high-quality meta memory priors.

#### 4.2.2. One Model for All MTSs

Diverse MTSs often exhibit varying characteristics, making it difficult for anomaly detection models to fully capture this complexity using a single parameter set. MVMT overcomes this by utilizing the MMA and memory generative modules to capture and store a range of dynamics, enabling the model to handle diverse MTSs with a unified set of parameters—a trait we refer to as the *one-for-all* property. This property can not only ensure MVMT to manage large MTS data but also adapt to new MTS with few observations, which will be proved in the experiment.

#### 4.2.3. Probability Modeling of Hidden Space and Generative Process

Previous deterministic transformer-based approaches may face challenges in modeling the variability present in highly structured MTS data, characterized by complex and strong dependencies across different timesteps. To overcome this, we introduce uncertainty into transformers by applying probabilistic modeling in both the hidden space and the generative process. Following similar arguments made by others [38], we propose that this probabilistic framework is particularly useful when the true data distribution is multimodal, which reflects the diverse nature of multivariate time-series data.

#### 4.2.4. Transformer for Time Series

Transformers have demonstrated efficiency in capturing complex, long-range temporal dependencies. MVMT applies transformer structure in both memory and inference modules, which enables our model to learn expressive memories and latent representations. Especially for multiple MTSs data, there are diverse patterns within them; traditional recurrent-structured networks may not be powerful enough to capture there dynamics, while our model can handle them efficiently with transformer structure.

#### 4.2.5. Beneficial to Process Large Data

MVMT is designed to efficiently handle large-scale MTS data. The learnable prior allows it to capture and store diverse characteristics within the data, while the mixture-distributed latent states help model various structural patterns and non-stationary temporal dependencies across different MTSs. By incorporating probabilistic modeling in the generative process, MVMT accounts for the variability present in complex MTS data. Additionally, the transformer architecture in both the inference and memory modules enhances its ability to capture diverse patterns. The ’one-for-all’ setting of MVMT can avoid the drawback of previous methods that the parameters grow exponentially facing to large data.

## 5. Model Training

To train the model end-to-end, we establish an objective function. This reconstruction-based approach captures the latent representation of the entire MTS. For MVMT, given the model parameters W, the marginal likelihood of the MTS dataset D is formulated as

$$p\left(D|W\right)=\int_{m_{a}}\int_{z}p\left(x|z,m_{a}\right)p\left(z|m_{a}\right)p\left(m_{a}\right)dm_{a}dz$$

Consistent with VAEs, MVMT aims to optimize by maximizing the evidence lower bound (ELBO) on the log marginal likelihood. The log marginal likelihood for MVMT is then expressed as

(16) $$\begin{array}{cc} \log p(x)= & E_{z∼q\left(z|m_{a},x\right)\;m_{a}∼q\left(m_{a}|M_{a},x\right)}\left[\log p\left(x\right)\right] \end{array}$$

Applying Bayes’ rule [39] for p(x), we incorporate the terms $q(z|m_{a},x)$, $p(z|m_{a})$, $p(m_{a}|M_{a})$, and $q(m_{a}|M_{a},x)$ to the logp(x) as

(17) $$\begin{array}{cc} \log p(x)= & \;E\;[\log\frac{p(x|z)p(z)}{p(z|x)}\frac{q(z|m_{a},x)}{q(z|m_{a},x)}\frac{p(z|m_{a})}{p(z|m_{a})}\\ \; & \frac{p(m_{a}|M_{a})}{p(m_{a}|M_{a})}\frac{q(m_{a}|M_{a},x)}{q(m_{a}|M_{a},x)}] \end{array}$$

Organizing Equation (17) can conclude

(18) $$\begin{array}{cc} \log p(x)= & \;E\;\left[\log p\left(x|z\right)\right]-E\;\left[\log\frac{q(z|m_{a},x)}{p(z|m_{a})}\right]\\ \; & -E\;\left[\log\frac{q(m_{a}|M_{a},x)}{p(m_{a}|M_{a})}\right]+E\;\left[\log\frac{q(m_{a}|M_{a},x)}{p(m_{a}|M_{a})}\right]\\ \; & +E\;\log\frac{q(z|m_{a},x)}{p(z|m_{a})}+E\;\left[\log\frac{p(z)}{p(z|x)}\right] \end{array}$$

Next, applying the Kullback–Leibler (KL) Divergence, defined as $D_{KL}\;\left(p∥q\right)$=E[log(p/q)], we can further reformulate Equation (18) as

(19) $$\begin{array}{cc} \log p(x)= & \;E\;\left[\log p\left(x|z\right)\right]-D_{KL}\;\left(q\left(z|m_{a},x\right)∥p\left(z|m_{a}\right)\right)\\ \; & -D_{KL}\;\left(q\left(m_{a}|M_{a},x\right)∥p\left(m_{a}|M_{a}\right)\right)\\ \; & +D_{KL}\;\left(q\left(m_{a}|M_{a},x\right)∥p\left(m_{a}|M_{a}\right)\right)\\ \; & +D_{KL}\;\left(q\left(z|m_{a},x\right)∥p\left(z|m_{a}\right)\right)\\ \; & +D_{KL}\;\left(p\left(z\right)∥p(z|x)\right) \end{array}$$

By applying Jensen’s inequality, we can derive the following expression for the ELBO:

(20) $$\begin{array}{cc} \log p(x)\geq & E\;\left[\log p\left(x|z\right)\right]-D_{KL}\;\left(q\left(z|m_{a},x\right)∥p\left(z|m_{a}\right)\right)\\ \; & -D_{KL}\;\left(q\left(m_{a}|M_{a},x\right)∥p\left(m_{a}|M_{a}\right)\right) \end{array}$$

Drawing inspiration from beta-VAE [40], we incorporate two hyperparameters, $\beta_{1}>0$ and $\beta_{2}>0$, which function as temperature coefficients. These coefficients are used in a ‘warm-up’ procedure, where we initially train the model focusing solely on reconstruction error. Gradually, over the first *N* training epochs, the KL divergence loss is introduced, applying an additional warm-up factor that increases linearly from 0 to 1 during training. This gradual introduction helps address the issue of posterior collapse, resulting in the final optimization target:

(21) $$\begin{array}{cc} L & =E_{q\left(z\right),q\left(m_{a}\right)}\;\left[\log p\left(x|z\right)\right]\\ & -\beta_{1}D_{KL}\;\left(q\left(z|m_{a},x\right)∥p\left(z|m_{a}\right)\right)\\ \; & -\beta_{2}D_{KL}\;\left(q\left(m_{a}|M_{a},x\right)∥p\left(m_{a}|M_{a}\right)\right) \end{array}$$

Unlike the conventional ELBO, which primarily focuses on the expected log-likelihood for maintaining reconstruction accuracy and the KL divergence for aligning the variational distribution of z with the prior, the ELBO of MVMT learns a set of meta memory prior (*M*) to guide latent variable $m_{a}$. The information stored in the memory units passes to z through a KL constraint between $m_{a}$ and z. The prior of z is obtained from the posterior probability of the $m_{a}$, so an additional KL is needed to give the $m_{a}$ prior. As demonstrated in Algorithm 1, the model parameters are optimized using stochastic gradient descent, allowing for an end-to-end training process. The algorithm implements an autoencoding variational inference strategy, employing an upward–downward scheme, to train and fine-tune the parameters essential for the MVMT model. Leveraging a pre-processed Multivariate Time Series (MTS) training dataset, this process encompasses encoding, memory management, latent variable inference, decoding, and iterative parameter updates, ensuring comprehensive model training and optimization.
**Algorithm 1** Upward–Downward Autoencoding Variational Inference for MVMT**Input**:The pre-processed MTS training dataset $D(x_{1:T})$.**Parameter**: The encoder parameters $\Psi =\{W_{xh_{0}},W_{h_{0}h_{1}},$$b_{h_{0}h1},W_{h_{0}h^{\prime }},$$b_{h_{0}h^{\prime }},$${\tilde{W}}_{m_{a}}^{\mu },$${\tilde{V}}_{m_{a}}^{\mu },$${\tilde{b}}_{m_{a}}^{\mu },$${\tilde{W}}_{m_{a}}^{\sigma },$${\tilde{V}}_{m_{a}}^{\sigma },$${\tilde{b}}_{m_{a}},$${\tilde{W}}_{z}^{\mu },$${\tilde{V}}_{z}^{\mu },$${\tilde{b}}_{z}^{\mu },$${\tilde{W}}_{z}^{\sigma },$${\tilde{V}}_{z}^{\sigma },$${\tilde{b}}_{z}\}$The decoder parameters $\Phi =\{W_{\mu }^{M_{a}},$$b_{\mu }^{M_{a}},$$W_{\sigma }^{M_{a}},$$b_{\sigma }^{M_{a}},$ $W_{\mu }^{m_{a}},$$b_{\mu }^{m_{a}},$ $W_{\sigma }^{m_{a}},$$b_{\sigma }^{m_{a}},$$W_{\mu }^{z},$$b_{\mu }^{z},$$W_{\sigma }^{z},$$b_{\sigma }^{z},\}$The meta memory units parameters M.**Output**:Parameters after training Ψ, Φ, M.  1:Set mini-banch size as *N* and hyperparameters $\beta_{1},\beta_{2}$;  2:Initialize the encoder parameters Ψ, decoder parameters Φ and meta memory units parameters M;  3:**while** epoch **do**  4:   Randomly select a mini-batch ${\left\{x_{1:T}^{i}\right\}}_{i=1}^{N}$ in $D(x_{1:T})$;  5:   Extract features $h=\{h_{0},h_{1},h^{\prime }\}$ using encoder;  6:   Inference the meta memory posterior $q(m_{a})$ through MMA (Equation (12));  7:   Inference the latent variable z through Equation (14);  8:   Input the latent variable to get the decoder result;  9:   Update the parameters(Ψ, Φ, M) by Equation (21);10:**end while**11:**return** Parameters after training Ψ, Φ, M.

## 6. Experimental Evaluation

This section begins by detailing the experimental setup, followed by a comprehensive evaluation of our model through various experiments.

### 6.1. Experiment Setup

#### 6.1.1. Datasets

Our experiments involve five datasets: (1) SMD [1], a 5-week dataset collected from a large internet company, consisting of 38 dimensions. (2) MSL (Mars Science Laboratory rover) and SMAP (Soil Moisture Active Passive satellite), which are publicly available NASA datasets [16] with 55 dimensions. Both datasets contain telemetry anomaly data sourced from Incident Surprise Anomaly (ISA) reports for spacecraft monitoring. (3) PSM [41], an internally collected dataset from multiple application server nodes at eBay, containing 26 dimensions. (4) SWaT [42], collected from 51 sensors monitoring a critical infrastructure system during continuous operation. (5) DND [43], a real-world multivariate KPI dataset from a major internet company in China, monitoring 12 websites with 36 KPIs per site. These websites offer various services, such as Video on Demand (VoD) and live streaming. The KPIs span roughly 1.5 months, with data points collected every 60 s. For each website, the first half of the KPIs are used for training and the second half for testing. Human operators confirmed the ground-truth anomalies in the DND testing data. Table 2 provides the basic statistical details of the datasets.

#### 6.1.2. Baselines

We compare our model with 19 baselines across various categories. For reconstruction-based models, we evaluate against InterFusion [44], BeatGAN [45], OmniAnomaly [1], and LSTM-VAE [46]. For density-estimation methods, we include DAGMM [47], MPPCACD [48], and LOF [49]. Clustering-based approaches like ITAD [50], THOC [51], and Deep-SVDD [52] are also considered. We further compare with autoregression-based models, including CL-MPPCA [53], LSTM [16], and VAR [54], as well as classic methods such as OC-SVM [55] and IsolationForest [56]. State-of-the-art deep learning models such as Anomaly Transformer [24] and InterFusion [44] are part of the evaluation, along with GmVRNN [30], a leading probabilistic model for anomaly detection, and TranAD [33], a recent anomaly detection method.

#### 6.1.3. Implementation Details

In our experiments, we implement a three-layer transformer similar to the Anomaly Transformer (without the minimax strategy from [24]), with hidden states of 128 dimensions serving as the encoder. The MLP used to map features $h_{0}$, $h_{1}$, and $h^{\prime }$ also has a dimension of 128. The MMA module comprises 15 memory units, each with a dimensionality of 128 across all datasets. Both the memory prior $m_{a}$ and latent variable z are set to a dimension of 128. We use hyperparameters $\beta_{1}$ and $\beta_{2}$ at 0.1 to balance the reconstruction and KL divergence terms across all datasets. The model is optimized using the Adam optimizer with a learning rate of 0.0002 and a batch size of 256. For the threshold in the POT method, we empirically set *p* to 0.003. Consistent with [51], a sliding window technique is applied to obtain sub-series, with a window size of 20 across all datasets. All experiments were carried out using PyTorch [57] on an NVIDIA RTX 3090 24 GB GPU.

#### 6.1.4. Metric and One-for-All Setting

We measure the model’s performance using Precision, Recall, and F1 score, as commonly used in similar studies [1,12,14]. The F1 score, in particular, offers a balanced view by combining both precision and recall. An anomaly is classified as a true positive if any point within the ground-truth anomaly segment is identified correctly [30]. In our experiments, we split each MTS dataset into two halves: the first half for training and the second half for testing. Please note that in the *one-for-one* experiment, we train individual models specifically for the MTS of each device, effectively preventing any negative impact resulting from mode variations across different devices. Conversely, the *one-for-all* experiment poses a greater challenge. In this task, we employ a single model for training and testing across all MTS from various devices.

### 6.2. Main Result

#### 6.2.1. Quantitative Comparison

We assessed our model’s performance on five public datasets and one real-world dataset, benchmarking it against a range of competitive methods. The results presented in Table 3 highlight that our model surpasses reconstruction-based methods like OmniAnomaly, density-estimation techniques such as MPPCACD, and even state-of-the-art deep learning models like Anomaly Transformer. Although TranAD [33], the best baseline anomaly detection method, is the *one-for-one* model, MVMT still exhibits 2.14% higher performance (F1) on average. Table 3 underscores the effectiveness of MVMT in time-series anomaly detection. Our model achieves superior performance across all five datasets, significantly reducing both false alarms and missed detections compared to other methods. This highlights MVMT’s potential for real-world applications where accuracy and reliability are crucial. Although MVMT achieves the best F1 score across all datasets (Table 3), the performance margin is noticeably smaller on datasets such as DND. These datasets exhibit relatively homogeneous normal patterns and limited temporal variability, allowing existing baselines to already model the underlying dynamics effectively. In such cases, the advantage of MVMT’s meta memory mechanism is less pronounced, as the need for capturing diverse temporal concepts is reduced. This observation provides insight into scenarios where MVMT may offer smaller gains despite overall superior performance.

#### 6.2.2. Ablation Study

**Figure 5:**
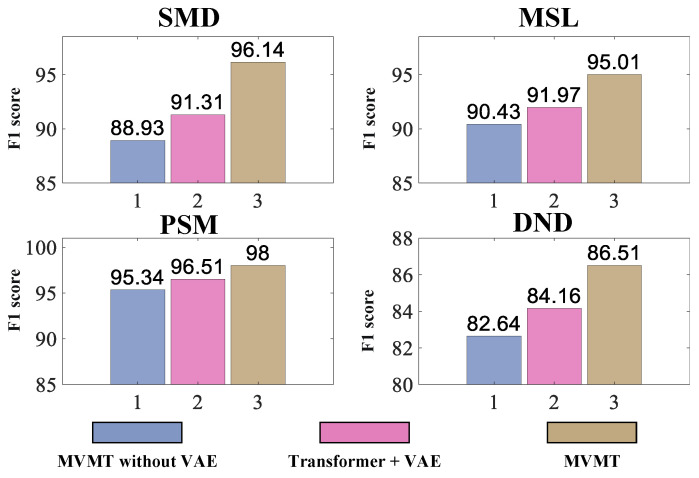
Ablation study of MVMT on four datasets.

To evaluate the influence of each component within our framework, we carried out ablation studies. The results of these studies are summarized in Figure 5. *MVMT-without-VAE* refers to using a basic MLP as a decoder, serving as the baseline in our model. *Transformer + VAE* represents a model where we introduce a single latent variable z without incorporating the $m_{a}$ component. Notably, *Transformer + VAE* exhibits an average performance 6.61% better than *MVMT-without-VAE* in Figure 5, demonstrating the effectiveness of employing probabilistic modeling. The complete version of our model, *MVMT*, integrates the proposed MMA model and the latent variable $m_{a}$. The MMA model, along with the latent variable $m_{a}$, achieves the best F1 score, surpassing *Transformer + VAE* by an average of 8.80% in Figure 5. This result underscores the significance of the MMA module, capable of capturing and storing various temporal dependencies within MTSs. Consequently, MVMT efficiently captures multiple patterns of MTSs with a single set of parameters. Furthermore, introducing the meta memory component in our model results in additional performance enhancements. Our experiments confirm that the diverse temporal information introduced by the MMA module significantly contributes to improving MTS anomaly detection.

#### 6.2.3. Meta Memory Attention

**Figure 6:**
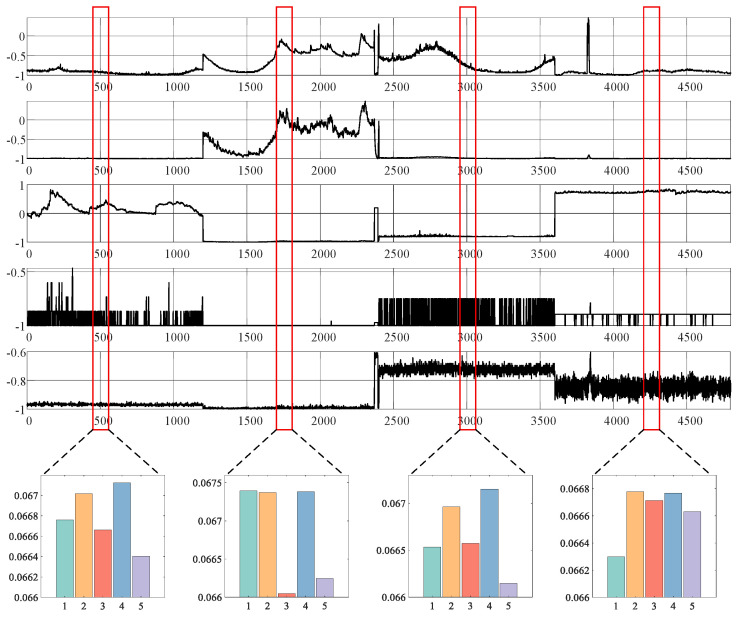
Visualization of the meta memory unit’s attention weights. Our memory module adeptly recalls relevant memories for different patterns. The upper section of the figure depicts segments of the MTS channels with four distinct patterns, while the lower section displays the attention weights for the corresponding positions, highlighted in red boxes.

In this paper, we aim to improve model performance by leveraging memory units to capture diverse temporal information within MTSs. Efficient retrieval of relevant memory units for various MTSs is crucial. To evaluate this, we analyzed the attention weights of the MMA, as shown in Figure 6. We selected five memory units at random and examined their attention weights. The upper part of the figure depicts MTS data with four distinct patterns, while the lower part displays a bar chart of the mean attention weights, outlined in the red box. The analysis in Figure 6 illustrates that the meta memory attention weights can be tailored to generate specific memories for different patterns in the MTS data. For instance, the yellow bar shows a relatively lower proportion for the first three patterns but becomes more prominent for the fourth pattern.

**Figure 7:**
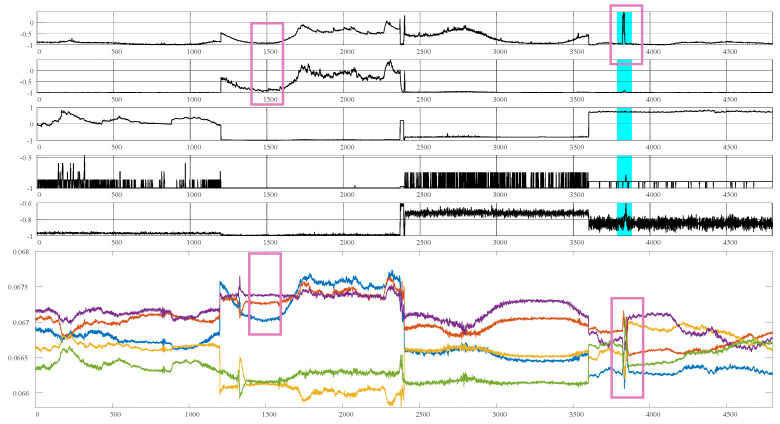
Visualization of attention weights in the meta memory units. Our memory module retrieves relevant memories for different patterns. The upper part of the figure depicts segments of MTS channels featuring four distinct patterns, whereas the lower part shows the associated attention weights.

In this section, we provide visual insights into the attention weights of the MMA. Figure 6 showcases the statistical properties across four different patterns of MTSs. Additionally, in Figure 7, we delve into observing subtle changes in a specific MTS pattern. For observation purposes, we randomly selected attention weights from five memory units. The upper section of the figure presents segments of the MTS data with four distinct patterns, while the lower section shows the corresponding attention weights.

In contrast to Figure 6, we visualize the attention weights for individual points within each MTS. As depicted in the purple box in Figure 7, the memory attention weights of MMA exhibit four distinct cases across the four patterns of MTS. However, within each pattern, MMA finely tunes the weights corresponding to specific time timesteps. This phenomenon illustrates that MVMT not only provides different memory priors for significant pattern changes but also adjusts the memory priors’ information based on slight variations in each MTS. Regarding abnormal states, in cases where there is no corresponding information in memory, the memory attention weights generate a distinctive response, enhancing the model’s performance in detecting anomalies.

#### 6.2.4. Information Capacity of MVMT

**Figure 8:**
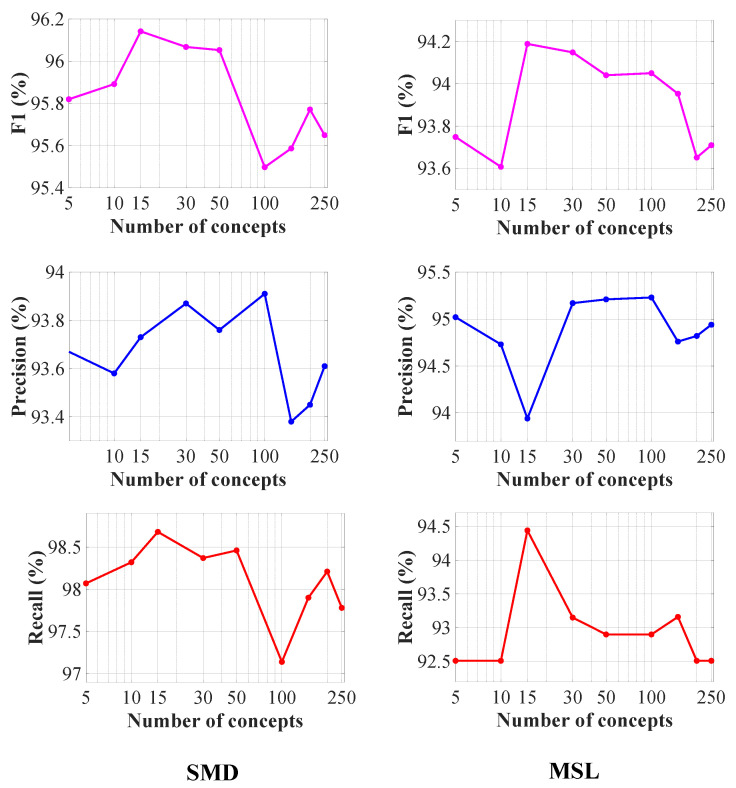
Visualization of how performance metrics change with varying numbers of memory units on the SMD and MSL datasets.

A key contribution of this paper is the introduction of a meta variational memory module aimed at learning diverse characteristics across multiple MTSs. The MMA is designed to store and recall historical MTS data, allowing MVMT to adapt quickly to new datasets. To examine how the number of memory units, denoted as *N*, affects model performance, we tested different *N* values, as shown in Figure 8. The results indicate that increasing *N* from 5 to 15 enhances the model’s capacity and performance. However, when *N* exceeds 15, the model may begin to overfit due to the accumulation of less relevant information.

#### 6.2.5. Adapt to New MTSs

In practical settings involving MTS data, such as CDN systems, there is often a need to adapt detection models to new MTSs with limited data availability. Previous methods face challenges in achieving this adaptation, especially in the *one-for-one* setting, as they often necessitate training individual models for new MTSs. This approach may falter when confronted with limited observations. Our proposed MVMT trains a unified set of parameters for all MTSs and harnesses global information using the MMA module. This capability empowers MVMT to adapt to new MTSs despite limited observations. To demonstrate the efficacy of MVMT in adapting to new MTSs, we designed two new experiments to evaluate the model’s performance in scenarios with minimal data.

For the experiments in this section, we allocated 80% of the MTSs as historical data and used the remaining 20% as new data. In practical settings, such as deploying a *one-for-one* anomaly detection model for new equipment, we might only have a limited number of MTS samples for training. Given that each sample takes about 60 s to acquire and setup time for the equipment is approximately 20 min, we trained the *one-for-one* models using the initial *i* samples (i∈1,5,10,20,200) from each new MTS and evaluated the model on the remaining samples.

For the *one-for-all* models (GmVRNN and MVMT), parameters were initially trained on historical MTS data and later fine-tuned using the first *i* samples (i∈1,5,10,20,200) from each new MTS. Table 4 outlines the detection performance across methods. The results show that *one-for-one* models, when trained on limited data, fail to accurately capture normal patterns, resulting in reduced performance. In contrast, *one-for-all* models leverage previously stored information from historical MTSs, leading to a noticeable improvement over the *one-for-one* approaches.

Additionally, to further validate the design of our methods tailored for the *one-for-all* setting, we conducted an additional experiment by pretraining the *one-for-one* models on the training data, similar to the pretraining strategy applied in the *one-for-all* models. As depicted in Table 4, although the *one-for-one* models perform relatively better due to the involvement of historical MTSs, they fail to capture and retain the diverse characteristics within multiple MTSs. In contrast, our proposed MVMT, equipped with multiple components for preserving and recalling historical information, exhibits significantly superior performance compared to the *one-for-one* models in this setting.

#### 6.2.6. Qualitative Analysis

**Figure 9:**
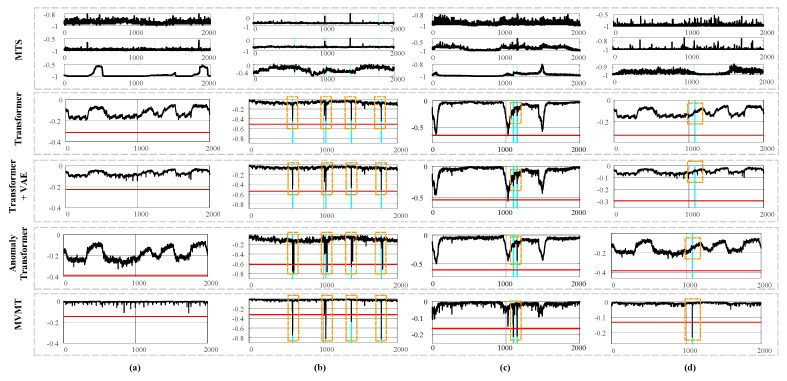
Anomaly score analysis on the SMD dataset. The blue-highlighted regions denote the ground-truth anomalies, and the red lines indicate the thresholds set by POT. The top row displays a segment of the MTS data, followed by the anomaly scores for MVMT, Anomaly Transformer, VAE, and a simple Transformer in the rows below. Panels (**a**–**d**) represent different time periods of the data.

Beyond quantitative analysis, we also conducted qualitative assessments. We compared the anomaly scores produced by MVMT, Anomaly Transformer, *MVMT without $m_{a}$*, and a standard Transformer, as shown in Figure 9. Deterministic models like Transformers tend to generate more fluctuating anomaly scores, as they do not account for the inherent stochasticity in MTS data. In contrast, probabilistic models, such as *MVMT without $m_{a}$*, provide smoother anomaly scores due to their more structured probabilistic design. However, dealing with certain situations using a simple Gaussian hidden variable remains challenging. Introducing a memory module into the anomaly detection model plays a vital role in improving performance. By incorporating diverse temporal information, the model can more accurately reconstruct normal MTS patterns, leading to enhanced detection accuracy and a lower false alarm rate. As shown in Figure 9a, the temporal diversity introduced by MMA allows MVMT to generate noticeably lower anomaly scores in normal data scenarios compared to other models. MMA’s retrieval of relevant memory is akin to referencing a vast historical dataset, helping MVMT produce smoother anomaly scores during normal conditions and more pronounced scores when anomalies occur. Figure 9b–d illustrate several cases where MVMT detects anomalies while other methods fail to do so. In (b), Transformer and *MVMT without $m_{a}$* fail to generate sufficiently high anomaly scores, leading to detection failure. The more sophisticated model designs of Anomaly Transformer and MVMT yield higher anomaly scores, successfully detecting anomalies. In (c), when two anomalies are proximate, other models may misconstrue them as usual or ignore one, whereas our proposed model can compare memory information to differentiate between these modes using MMV. In (d), the model easily disregards inconspicuous anomalies as noise, while MVMT can sensitively detect them.

#### 6.2.7. Time Efficiency

In line with previous studies [30], we assessed the time efficiency of various methods, including our MVMT, transformer-based models like TranAD and Anomaly Transformer, and RNN-based approaches such as GmVRNN and LSTM-VAE. The results, shown in Table 5, indicate that MVMT had notably shorter training and testing times compared to the other methods when using the same GPUs. All models were able to detect anomalies in less than a second per sample, which is beneficial given the 60 s interval between data points. This suggests that these methods are well-suited for real-time anomaly detection.

#### 6.2.8. Number of Parameters

**Figure 10:**
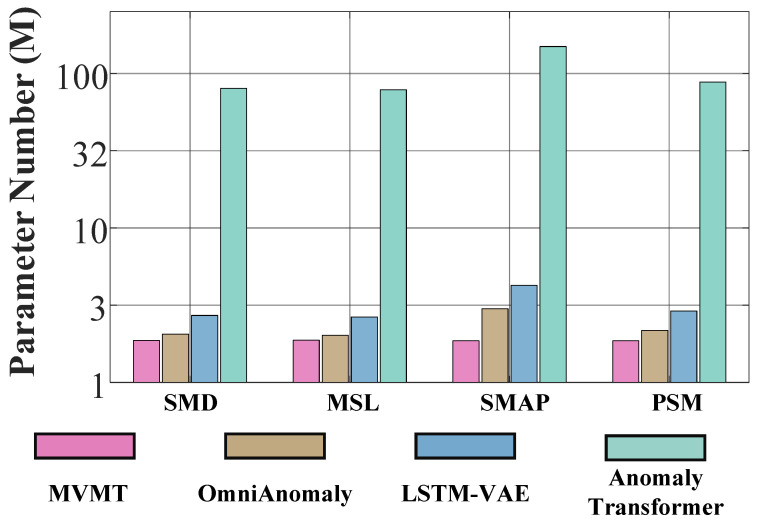
Comparison of model parameters.

We analyzed and compared the number of model parameters across various methods, as shown in Figure 10. For this analysis, we selected LSTM-VAE, OmniAnomaly and Anomaly Transformer as baselines, representing typical RNN-based and Transformer-based methods, respectively. Notably, the parameter count of OmniAnomaly is considerably smaller than that of Anomaly Transformer due to the complexity of the transformer structure. However, with the incorporation of a mixture-distributed latent variable and a meta memory module, our proposed MVMT can efficiently account for the diverse characteristics within various MTSs. This capability allows MVMT to effectively model all MTSs using a single set of parameters, resulting in superior performance compared to previous methods, even surpassing RNN-based methods, with significantly fewer parameters.

## 7. Conclusions

To address anomaly detection in large-scale Multivariate Time Series (MTS), we propose the Meta Variational Memory Transformer (MVMT), which integrates self-attention with a meta memory module and a memory-guided probabilistic generative framework to effectively capture, organize, and utilize temporal concepts across diverse MTSs. The upward–downward autoencoding inference scheme further enhances posterior estimation through the integration of bottom-up likelihood and top-down priors. Across five public datasets and one real-world dataset, MVMT consistently attains the top F1 performance among all compared methods. Beyond accuracy, the ability to apply a single set of parameters to heterogeneous MTS collections highlights MVMT’s practicality and scalability for real-world online anomaly detection. These results collectively demonstrate the effectiveness and applicability of MVMT in large-scale MTS environments.

## Figures and Tables

**Table 1:** Symbol description for MVMT.

Encoder		Decoder	
Symbols	Description	Symbols	Description
$W_{xh_{0}}$	The model parameter symbol of the Anomaly Transformer	$W_{\mu }^{M_{a}}$	MLP weights mapped from $M_{a}$ to the mean (μ) of $m_{a}$
$W_{h_{0}h_{1}}$	The MLP weights of feature $h_{0}$ to $h_{1}$.	$b_{\mu }^{M_{a}}$	MLP bias mapped from $M_{a}$ to the mean (μ) of $m_{a}$
$b_{h_{0}h1}$	The MLP bias of feature $h_{0}$ to $h_{1}$.	$W_{\sigma }^{M_{a}}$	MLP weights mapped from $M_{a}$ to the variance (σ) of $m_{a}$
$W_{h_{0}h^{\prime }}$	The MLP weights of feature $h_{0}$ to $h^{\prime }$.	$b_{\sigma }^{M_{a}}$	MLP bias mapped from $M_{a}$ to the variance (σ) of $m_{a}$
$b_{h_{0}h^{\prime }}$	The MLP bias of feature $h_{0}$ to $h^{\prime }$.	$W_{\mu }^{m_{a}}$	MLP weights mapped from $m_{a}$ to the mean (μ) of *z*.
${\tilde{W}}_{m_{a}}^{\mu }$	The MLP weights that approximate the mean (μ) of $m_{a}$ with $M_{a}$	$b_{\mu }^{m_{a}}$	MLP bias mapped from $m_{a}$ to the mean (μ) of *z*.
${\tilde{V}}_{m_{a}}^{\mu }$	The MLP weights that approximate the mean (μ) of $m_{a}$ with $h_{1}$	$W_{\sigma }^{m_{a}}$	MLP weights mapped from $m_{a}$ to the variance (σ) of *z*.
${\tilde{b}}_{m_{a}}^{\mu }$	The MLP bias that approximate the mean (μ) of $m_{a}$	$b_{\sigma }^{m_{a}}$	MLP bias mapped from $m_{a}$ to the variance (σ) of *z*.
${\tilde{W}}_{m_{a}}^{\sigma }$	The MLP weights that approximate the variance (σ) of $m_{a}$ with $M_{a}$	$W_{\mu }^{z}$	MLP weights mapped from *z* to the mean (μ) of *x*.
${\tilde{V}}_{m_{a}}^{\sigma }$	The MLP weights that approximate the variance (σ) of $m_{a}$ with $h_{1}$	$b_{\mu }^{z}$	MLP bias mapped from *z* to the mean (μ) of *x*.
${\tilde{b}}_{m_{a}}$	The MLP bias that approximate the variance (σ) of $m_{a}$	$W_{\sigma }^{z}$	MLP weights mapped from *z* to the variance (σ) of *x*.
${\tilde{W}}_{z}^{\mu }$	The MLP weights that approximate the mean (μ) of *z* with $M_{a}$	$b_{\sigma }^{z}$	MLP bias mapped from *z* to the variance (σ) of *x*.
${\tilde{V}}_{z}^{\mu }$	The MLP weights that approximate the mean (μ) of *z* with $h_{1}$		
${\tilde{b}}_{z}^{\mu }$	The MLP bias that approximate the mean (μ) of *z*		
${\tilde{W}}_{z}^{\sigma }$	The MLP weights that approximate the variance (σ) of *z* with $M_{a}$		
${\tilde{V}}_{z}^{\sigma }$	The MLP weights that approximate the variance (σ) of *z* with $h_{1}$		
${\tilde{b}}_{z}$	The MLP bias that approximate the variance (σ) of *z*		

**Table 2:** Basic statistics of datasets.

Dataset	SMD	MSL	SMAP	PSM	DND	SWaT
Dimension	38	55	55	25	32	51
Window	20	20	20	20	20	20
Training	708,405	58,317	135,181	105,984	344,843	496,800
Test (labeled)	708,420	73,729	427,617	87,841	344,843	449,919
Anomaly ratio (%)	4.1	10.7	13.13	27.8	3.44	11.98

**Table 3:** We report the quantitative performance of different methods across five public datasets and one real-world dataset. The metrics used include Precision (P), Recall (R), and F1 score (in percentages), with the F1 score representing the harmonic mean of precision and recall. Higher values across these metrics indicate superior performance. Boldface indicates the best F1 score among all compared methods.

Dataset	SMD			MSL			PSM			SMAP			DND			SWaT		
Metric	P	R	F1	P	R	F1	P	R	F1	P	R	F1	P	R	F1	P	R	F1
OC-SVM	44.34	76.72	56.19	59.78	86.87	70.82	62.75	80.89	70.67	53.85	59.07	56.34	68.23	70.36	69.28	45.39	49.22	47.23
IsolationForest	42.31	73.29	53.64	53.94	86.54	66.45	76.09	92.45	83.48	52.39	59.07	55.53	69.63	74.21	71.85	49.29	44.95	47.02
LOF	56.34	39.86	46.68	47.72	85.25	61.18	57.89	90.49	70.61	58.93	56.33	57.60	70.24	74.66	72.38	72.15	65.43	68.62
Deep-SVDD	78.54	79.67	79.10	91.92	76.63	83.58	95.41	86.49	90.73	89.93	56.02	69.04	73.09	79.02	75.94	80.42	84.45	82.39
DAGMM	67.30	49.89	57.30	89.60	63.93	74.62	93.49	70.03	80.08	86.45	56.73	68.51	72.72	77.67	75.11	89.92	57.84	70.40
MMPCACD	71.20	79.28	75.02	81.42	61.31	69.95	76.26	78.35	77.29	88.61	75.84	81.73	71.52	75.75	73.57	82.52	68.29	74.73
VAR	78.35	70.26	74.08	74.68	81.42	77.90	90.71	83.82	87.13	81.38	53.88	64.83	72.92	77.72	75.24	81.59	60.29	69.34
LSTM	78.55	85.28	81.78	85.45	82.50	83.95	76.93	89.64	82.80	89.41	78.13	83.39	73.52	79.67	76.47	86.15	83.27	84.69
CL-MPPCA	82.36	76.07	79.09	73.71	88.54	80.44	56.02	99.93	71.80	86.13	63.16	72.88	73.12	78.71	75.81	76.78	81.50	79.07
ITAD	86.22	73.71	79.48	69.44	84.09	76.07	72.80	64.02	68.13	82.42	66.89	73.85	73.87	75.43	74.64	63.13	52.08	57.08
LSTM-VAE	75.76	90.08	82.30	85.49	79.94	82.62	73.62	89.92	80.96	92.20	67.75	78.10	75.10	79.05	77.02	76.00	89.50	82.20
BeatGAN	72.90	84.09	78.10	89.75	85.42	87.53	90.30	93.84	92.04	92.38	55.85	69.61	76.64	80.18	78.37	64.01	87.46	73.92
OmniAnomaly	83.68	86.82	85.22	89.02	86.37	87.67	88.39	74.46	80.83	92.49	81.99	86.92	79.47	82.37	80.90	81.42	84.30	82.83
InterFusion	87.02	85.43	86.22	81.28	92.70	86.62	83.61	83.45	83.52	89.77	88.52	89.14	78.21	85.34	81.62	80.59	85.58	83.01
THOC	79.76	90.95	84.99	88.45	90.97	89.69	88.14	90.99	89.54	92.06	89.34	90.68	80.97	83.45	82.19	83.94	86.36	85.13
GmVRNN	96.07	91.23	93.56	90.81	92.10	91.41	95.62	98.36	96.97	96.51	94.54	95.51	83.80	87.67	85.58	90.11	94.69	92.34
Anomaly Transformer	89.40	95.45	92.33	92.09	95.15	93.59	96.91	98.90	97.89	94.13	99.40	96.69	82.13	86.31	84.16	91.55	96.73	94.07
TranAD	92.62	99.74	96.05	90.38	99.99	94.94	95.36	98.65	96.97	80.43	99.99	89.15	82.59	86.60	84.54	97.60	69.97	81.51
Ours	93.73	98.68	**96.14**	91.89	98.37	**95.01**	96.65	99.39	**98.00**	94.26	99.28	**96.70**	84.58	88.54	**86.51**	94.08	97.58	**95.79**

**Table 4:** The quantitative results for *adapting to new MTSs* across three public datasets are shown. These results were obtained by training the models with a limited amount of new MTS data and then testing their performance directly on this data. Boldface indicates the best F1 score among all compared methods.

Methods	Dataset	SMD					MSL					SMAP				
	Data Number	1	5	10	20	200	1	5	10	20	200	1	5	10	20	200
	Metric	F1	F1	F1	F1	F1	F1	F1	F1	F1	F1	F1	F1	F1	F1	F1
**Random** **Initialization**	DAGMM	43.11	44.16	44.87	44.02	63.38	50.66	50.52	51.36	51.45	65.15	53.38	53.26	54.89	60.16	65.68
	MMPCACD	54.40	55.49	55.63	56.21	67.05	51.31	51.94	52.57	52.75	65.08	52.90	52.76	56.01	61.05	65.87
	VAR	54.40	54.29	54.65	55.75	66.39	53.99	53.02	53.60	53.82	66.31	55.29	55.18	56.36	62.90	66.00
	LSTM	56.17	56.68	56.87	56.89	67.76	60.58	60.62	61.29	61.37	69.59	56.73	56.57	57.58	61.98	67.74
	CL-MPPCA	57.00	57.05	57.17	57.87	68.14	59.67	59.34	60.30	60.69	70.08	58.76	58.43	58.28	65.55	68.36
	ITAD	56.36	56.08	56.51	56.90	66.93	62.98	62.71	63.17	63.35	69.50	62.74	62.71	63.85	66.95	68.85
	LSTM-VAE	58.38	58.74	58.08	59.48	69.15	61.89	61.10	61.90	62.16	71.05	61.61	61.23	62.19	67.41	69.16
	BeatGAN	61.30	61.18	61.04	62.02	68.73	63.09	63.57	64.23	65.88	74.30	61.49	61.18	61.72	66.89	71.10
	OmniAnomaly	64.29	65.15	63.53	62.05	68.31	64.36	64.43	65.14	68.77	73.80	63.13	65.55	65.61	68.72	70.27
	InterFusion	63.68	62.66	62.37	62.28	69.83	63.60	63.56	64.32	65.83	62.16	63.50	63.34	63.95	68.79	72.44
	THOC	64.41	63.47	63.12	63.94	71.01	64.33	64.13	64.22	66.00	74.65	62.24	62.46	63.92	70.66	75.22
	Anomaly Transformer	64.24	65.09	71.85	76.98	81.11	65.16	64.99	68.52	70.46	77.02	63.82	66.72	67.34	71.45	78.88
	TranAD	66.28	67.58	72.26	77.01	81.54	67.66	66.26	69.83	72.74	78.94	64.27	67.24	68.73	72.65	80.78
**Pretraining with** **History MTSs**	DAGMM	72.08	72.55	72.82	73.04	73.39	68.47	67.71	68.28	68.68	69.17	70.81	71.04	71.66	71.94	72.07
	MMPCACD	73.15	72.90	73.90	73.31	73.11	69.99	69.68	70.34	70.57	70.04	72.95	73.01	73.05	73.25	73.44
	VAR	74.54	75.01	74.11	74.78	74.10	70.88	71.14	71.09	71.67	71.96	71.87	72.16	72.67	72.38	72.56
	LSTM	73.77	73.61	73.78	73.92	73.70	71.79	71.32	72.01	72.17	71.94	74.07	74.57	74.44	73.76	74.81
	CL-MPPCA	75.23	75.18	74.92	75.31	75.87	73.57	72.99	73.51	73.36	73.12	76.50	75.96	76.15	76.74	76.64
	ITAD	77.03	76.85	77.76	77.67	76.99	77.08	77.00	77.49	77.18	77.64	78.80	78.89	79.31	78.82	79.34
	LSTM-VAE	80.65	80.73	80.13	81.68	81.51	76.19	76.31	76.47	76.20	77.02	77.92	77.83	77.96	78.19	78.50
	BeatGAN	82.90	83.02	83.08	82.93	82.57	79.67	79.66	80.56	80.21	80.84	81.66	81.61	82.12	82.50	82.36
	OmniAnomaly	85.40	85.42	85.78	86.19	85.83	81.12	80.58	82.13	81.86	81.64	84.28	84.71	84.61	84.97	85.49
	InterFusion	84.60	84.73	84.74	84.85	85.09	82.09	81.87	82.28	82.46	83.00	86.70	86.80	87.26	87.27	87.12
	THOC	86.01	86.05	86.24	86.62	86.54	81.56	81.55	81.38	82.73	82.86	88.95	89.28	88.92	88.54	88.95
	Anomaly Transformer	91.58	91.92	91.46	91.78	91.69	80.65	82.87	82.15	83.55	84.01	93.32	93.89	93.51	94.01	94.11
	TranAD	91.96	91.69	91.57	91.87	91.94	80.85	81.44	82.42	84.08	84.12	93.01	94.03	93.62	94.35	94.37
**One-For-All**	GmVRNN	91.03	89.51	90.01	90.78	90.34	81.68	81.15	82.12	81.59	81.22	93.47	93.41	93.01	92.21	94.12
	Ours	**93.31**	**92.85**	**93.90**	**93.78**	**93.08**	**89.22**	**89.90**	**89.97**	**89.03**	**90.30**	**95.27**	**95.22**	**95.26**	**95.47**	**95.61**

**Table 5:** Training and testing time of different methods.

Methods	Average Training Times per Epoch (s)					Testing Times per Sample (s × 10^5^)				
	SMD	MSL	SMAP	PSM	DND	SMD	MSL	SMAP	PSM	DND
LSTM-VAE	26.71	3.88	6.22	40.29	37.59	8.37	14.34	8.90	8.51	9.76
OmniAnomaly	276.97	21.31	27.05	243.24	230.81	35.08	67.52	40.39	40.78	44.82
GmVRNN	25.20	2.67	5.04	37.96	35.26	7.02	13.54	8.17	8.26	8.89
Anomaly Transformer	28.80	2.64	5.40	39.67	6.15	6.51	11.34	7.21	7.32	7.95
TranAD	43.76	5.57	10.55	63.58	60.30	9.17	17.63	10.49	10.66	11.71
Ours	25.28	2.03	4.97	36.73	34.84	5.30	10.07	6.12	6.16	6.71

## Data Availability

The original contributions presented in this study are included in the article. Further inquiries can be directed to the corresponding author(s).
